# Gender differences in HIV risk behaviors in individuals recently released from prison: results of a pilot study

**DOI:** 10.1186/s40352-014-0014-y

**Published:** 2015-03-31

**Authors:** Gefei A Zhu, Nathan Birnbaum, Amy Carroll-Scott, Linda Evans, Lynn E Fiellin, Emily A Wang

**Affiliations:** 1 Stanford University School of Medicine, 291 Campus Dr., Stanford, CA 94305 USA; 2 Department of Internal Medicine, Yale University School of Medicine, 367 Cedar St. Harkness Building A, Ste 304, New Haven, CT 06510 USA; 3 Drexel University School of Public Health, Nesbitt Hall, 3215 Market St., Philadelphia, PA 19104 USA; 4 All of Us or None, 1904 Franklin St., Oakland, CA 94612 USA; 5 Department of Internal Medicine, Yale University School of Medicine, 333 Cedar St., PO BOX 208056, New Haven, CT 06520-8056 USA; 6 Department of Internal Medicine, Yale University School of Medicine, 367 Cedar St Harkness Building A, New Haven, CT 06510 USA

**Keywords:** Prison, Human immunodeficiency virus, Risk behaviors, Incarceration, Gender disparities

## Abstract

**Background:**

Individuals recently released from prison engage in risky behaviors that predispose them to contracting HIV. Women may be at increased risk in the immediate period post-release, given higher rates of poverty, food insecurity, and substance dependence and lower educational attainment compared with men.

**Methods:**

We describe gender differences in HIV risk behaviors using validated measures and assess potential mediators of this relationship using data from a cross-sectional study of 109 individuals recently released from prison.

**Results:**

Women had higher rates of HIV drug-related risk behaviors compared with men (mean score 2.72 vs. 0.068; p = .003) and HIV sex-related risk behaviors (mean score 4.32 vs. 2.31; p = .016). Women also had higher mean incomes and severity of drug and alcohol use compared with men, but equally high rates of food insecurity and low levels of AIDS knowledge. In multivariate analysis, the relationship between gender and HIV drug-related and sex-related risk behaviors was attenuated by a greater monthly income ([drug] adjusted β 0.82, 95% CI -1.02 – 2.66, p = 0.38; [sex] adjusted β 0.75, 95% CI -1.04 – 2.54, p = 0.41), as well as severity of drug use ([drug] adjusted β 0.79, 95% CI -0.55 – 2.13, p = 0.24; [sex] adjusted β 0.09, 95% CI -1.17 – 1.35, p = 0.89).

**Conclusions:**

Women had higher rates of HIV risk behaviors compared with men post-release. Gender specific interventions may be useful in reducing risky drug-related and sex-related behaviors in the period immediately following release.

## Background

Approximately eight million individuals are released from U.S correctional facilities each year and are at increased risk for HIV. Previous studies of this population describe frequent engagement in drug- and sex-related risk behaviors immediately after release (J. Adams et al. 2011; Binswanger et al. 2012; Morrow and Project START Study Group 2009), which contribute to the increased all-cause mortality and risk of acquiring and/or transmitting HIV post release. (Binswanger et al. 2007; Morrow and Project START Study Group 2009).

The majority of studies on risk behaviors among criminal justice-involved populations have focused either exclusively on men or women. (Fogel and Belyea 1999; Ricks et al. 2014; Knittel et al. 2013; MacGowan et al. 2003). However, the few comparative studies suggest there are significant differences in risk behaviors by gender. Some studies found that men engage in higher rates of drug-related HIV risk behavior in comparison with women; others showed that women are more likely to engage in transactional and unprotected sex. (Oser et al. 2006; Binswanger et al. 2014; Khan et al. 2008; L. M. Adams et al. 2013). These studies have primarily focused on individual risk behaviors, with few exploring how specific factors mediate increased risk for HIV by gender. (Binswanger et al. 2014; Khan et al. 2008; L. M. Adams et al. 2013).

The present study sought to describe gender differences in overall HIV risk behaviors among individuals recently released from prison using a validated, composite measure of drug- and sex-related risk behaviors. We additionally sought to explore whether socioeconomic status, food insecurity, AIDS knowledge, or severity of substance use would mediate the relationship between gender and HIV risk behaviors. We hypothesized that recently-released women would report higher rates of drug-and sex-related HIV risk behaviors compared with men.

## Methods

This is a secondary data analysis of a pilot study designed using a community based participatory research approach (Israel et al.) to explore the association between food insecurity and HIV risk behaviors among individuals recently released from prison within one calendar year of the interview. (Wang et al. 2013) Trained interviewers in San Antonio, TX, San Francisco, CA, and New Haven, CT, with a history of incarceration, recruited respondents from local reentry centers, halfway houses, homeless shelters, outdoor parks, or locations where individuals released from prison congregate, and administered a survey. Respondents were verbally consented and provided with $10 grocery vouchers for participation. The Yale University School of Medicine Human Investigation Committee approved this study.

The independent variable for this current study was self-reported gender. The dependent variables were engagement in drug-related risk behaviors (such as sharing and/or reusing needles) and sex-related risk behaviors (such as having multiple partners, not using condoms, or engaging in transactional sex) in the 30 days prior to interview, as assessed by the HIV Risk-Taking Behavior Scale-PLUS (HRBS-Plus) (Darke et al. 1991). Other covariates of interest included addiction severity, assessed by the ASI-Lite (McLellan et al. 1980), food security assessed using a modified USDA Food Security Module, (United States Department of Agriculture Economic Research Service), and AIDS knowledge as measured using a validated 40-item test (Kelly et al. 1989).

The association between gender and HRBS-Plus scores and other covariates of interest were compared using Student’s t tests for continuous variables or chi-squared tests or Fisher’s exact tests for categorical variables. Linear regression models were constructed with HRBS drug and sex sub-scores as the dependent variables and gender as the independent variable; models were adjusted for demographic variables followed by potential mediators selected *a priori* that were associated with both the independent and dependent variables at a level of statistical significance of p < 0.05 (Baron and Kenny 1986). Regression coefficients were reported with 95% confidence intervals. All analyses were conducted in STATA 12 (StataCorp LP; College Station, TX).

## Results

We enrolled 110 individuals out of 113 eligible approached subjects for an overall participation rate of 97%. Out of 109 individuals included in the final analyses, 50 (46%) were women. There were no statistically significant differences between men and women released from prison in terms of age, race, educational attainment, housing, or employment (Table 1).

**Table 1:** **Sociodemographic, clinical and behavioral characteristics by gender**

**Variable**	**Women, N = 50 N (%) or median (IQR) or mean (SD)**	**Men, N = 59 N (%) median (IQR) mean (SD)**	**P-value**
*Sociodemographic variables*			
Mean age	36.8 (8.4)	38.1 (12)	0.53
Race*			0.26
White	10 (20)	19 (33)	
Black	28 (57)	31 (53)	
Other	11 (22)	8 (14)	
Hispanic	5 (10)	9 (15)	0.57
Employed	3 (6)	7 (12)	0.34
Marital status			
Never married, or living alone	30 (60)	41 (71)	
Married, or living with a partner	6 (12)	9 (16)	0.19
Separated, divorced, or widowed	14 (28)	8 (14)	
Education			
Less than high school	18 (36)	19 (33)	
High school degree or GED	20 (40)	30 (52)	0.39
Some college	12 (24)	9 (16)	
Number of minor children	1.59 (1.50)	1.13 (1.32)	0.26
Immediate housing post-release			0.49
Homeless	20 (40)	23 (39)	
Transitional housing	10 (20)	15 (25)	
Family/Friends	16 (32)	19 (32)	
Own apartment/house	4 (8)	1 (0.02)	
Current housing			1.00
Homeless	4 (8)	2 (0.03)	
Transitional housing	1 (2)	0 (0)	
Family/Friends	3 (6)	1 (0.02)	
Own apartment/house	3 (6)	1 (0.02)	
Monthly income	845 (843)	308 (262)	0.001
Low Income (≤130% FPL)**	36 (77)	55 (100)	<0.001
Sex work	6 (12)	3 (5)	0.30
*Incarceration History Variables*			
Median days since most recent release	121 (64, 285)	126 (74, 185)	0.32
Times incarcerated, lifetime			
1	16 (32)	18 (32)	
2–5	17 (34)	15 (26)	0.25
>5	13 (26)	23 (40)	
Don’t know	4 (8)	1 (2)	
Total years incarcerated, lifetime			
<1	7 (14)	2 (4)	
1–10	36 (72)	34 (62)	0.016
>10	7 (14)	19 (35)	
On parole or probation	43 (86)	52 (91)	0.39
Most recent crime was drug-related	23 (47)	31 (58)	0.24
*Food security variables*			
Food insecure	43 (86)	56 (95)	0.18
Severely food insecure	22 (44)	18 (31)	0.15
AIDS knowledge score	20.3 (6.23)	21.6 (5.19)	0.26
*Drug and alcohol use variables*			
Overall drug use	29 (58)	25 (42)	0.10
Intravenous drug use	11 (22)	1 (2)	0.001
ASI-lite drug score	0.11 (0.16)	0.04 (0.05)	0.002
ASI-lite alcohol score	0.14 (0.20)	0.08 (0.13)	0.06
*Risk-taking behavior variables*			
HRBS-plus composite score	7.04 (11.48)	2.37 (2.57)	0.003
HRBS-plus drug subscore	2.72 (6.62)	0.068 (0.52)	0.003
HRBS-plus sex subscore	4.32 (5.69)	2.31 (2.53)	0.016

*Respondents could report multiple races and ethnicities, so this may not add up to 100%.

**Calculated for a single person household in 2013. This amount is $1,210 per month in income.

Further, we did not identify any differences in rates of food insecurity (86% vs. 95%; p = 0.18) or AIDS knowledge (mean score 20.3 vs. 21.6; p = 0.26) by gender. Women had a greater income compared with men ($845 ± 843 vs. $308 ± 262, p = 0.001). Despite similar rates of drug use (58% vs. 42%, p = 0.10), a higher proportion of women reported using intravenous drugs (22% vs. 2%, p = 0.001). Women were more likely to report higher severity of drug use (0.11 vs. 0.04, p = 0.002) but not alcohol use (0.14 vs. 0.08, p = 0.06).

Women were more likely to engage in drug-related HIV risk behaviors (mean score 2.72 vs. 0.068; p = 0.003) and sex-related HIV risk behaviors (mean score 4.32 vs. 2.31; p = 0.016). In multivariate analyses, adjustment for age and race did not attenuate the impact of gender on drug-related (unadjusted β 2.65, 95% 0.94–4.37; adjusted β 2.72, 95% CI 0.93–4.5, Table 2) or sex-related risk behaviors (unadjusted β 2.01, 95% 0.38–3.65; adjusted β 1.82, 95% CI 0.18–3.46). We next adjusted for monthly income and ASI-Lite drug scores separately, the only covariates associated with both the independent and dependent variables of interest. Inclusion of monthly income attenuated the association between gender and drug-related risk behaviors (adjusted β 0.82, 95% CI -1.02–2.66) and sex-related risk behaviors (adjusted β 0.75, 95% CI -1.04–2.54). Inclusion of the ASI-Lite drug score attenuated the relationship between gender and drug-related (adjusted β 0.79, 95% CI -0.55 – 2.13) and sex-related behaviors (adjusted β 0.09, 95% CI -1.17 – 1.35).

**Table 2:** **Gender differences in hiv risk behaviors, unadjusted and adjusted models**

	**Model 1: unadjusted model (β [95% CI], p-value)**	**Model 2: adjusted for age, race (β [95% CI], p-value)**	**Model 3: additionally adjusted for monthly income (β [95% CI], p-value)**	**Model 4: additionally adjusted for ASI-Lite drug score (β [95% CI], p-value)**
Men	Referent	Referent	Referent	Referent
Women	HRBS-plus drug score: 2.65 (0.94 – 4.37), p = 0.003	HRBS-plus drug score: 2.72 (0.93 – 4.50), p = 0.003	HRBS-plus drug score: 0.82 (-1.02 – 2.66), p = 0.38	HRBS-plus drug score: 0.79 (-0.55 – 2.13), p = 0.24
	HRBS-plus sex score: 2.01 (0.38 – 3.65), p = 0.02	HRBS-plus sex score: 1.82 (0.18 – 3.46), p = 0.03	HRBS-plus sex score: 0.75 (-1.04 – 2.54), p = 0.41	HRBS-plus sex score: 0.09 (-1.17 – 1.35), p = 0.89

**Figure 1:**
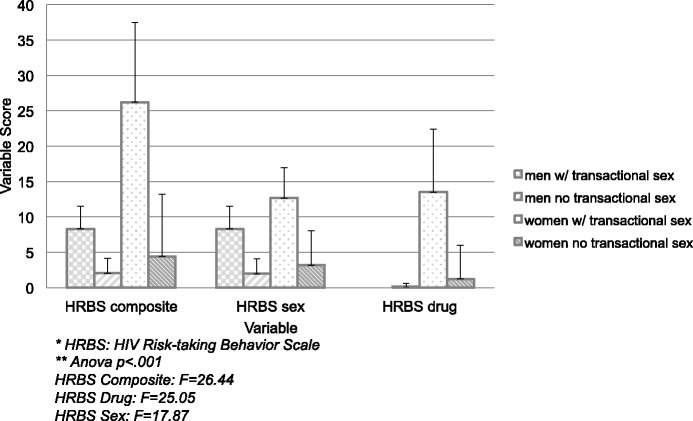
**Gender differences in HIV risk behavior by engagement in transactional sex.**

Given that women reported a greater monthly income and increased engagement in risky behaviors compared with men, we investigated whether these findings could be attributable to higher rates of transactional sex among women. Women did not have higher rates of engaging in transactional sex than men (12% vs. 5%, p = 0.30), but women who engaged in transactional sex had higher composite HRBS scores compared with men who engage in transactional sex (26.2 ± 11.3 vs. 8.3 ± 3.2, p = 0.036), driven by drug-related HIV risk behaviors (13.5 ± 8.9 vs. 0, p = 0.04, Figure 1). Likewise, individuals who engaged in transactional sex had a higher mean income compared with those who did not ($1622 ± 1039 vs. $465 ± 531, p < 0.0001). After excluding individuals who engage in transactional sex from the analyses, women still had a higher HIV risk behavior score compared with men (unadjusted β 2.38, 95% CI -0.36–4.79, p = 0.05).

## Discussion

We found that women recently released from prison reported increased engagement in drug- and sex-related risk behaviors compared with their male counterparts, in spite of similar knowledge of the risk factors for acquiring AIDS, rates of food insecurity, educational attainment and employment. Women released from prison had high severity of drug use, which increased the likelihood of engaging in high-risk behaviors. Women in our study, especially those engaging in transactional sex, reported higher rates intravenous drug use, though rates of overall drug use were not significantly different by gender. These findings suggest, like many prior studies, that strategies which acknowledge the parallel risk of illicit drug use and transactional sex among women, may also have an impact on reducing HIV transmission. (Binswanger et al. 2009; Fazel et al. 2006).

Unlike past studies, we did not find statistically significant differences in the rates of transactional sex by gender. (Binswanger et al. 2014; Khan et al. 2008). Instead, we found that women who were engaging in transactional sex had higher overall HIV risk behavior scores compared with men engaging in transactional sex. Moreover, individuals who engaged in transactional sex had significantly higher mean income compared with those who did not engage in transactional sex, perhaps explaining why income attenuated the association between gender and HIV risk behavior scores in the multivariate analysis. However, even after excluding participants who engaged in transactional sex, women still reported increased HIV risk behaviors compared with men. This finding suggests that women’s risk was not solely attributable to engaging in transactional sex, but rather were related to other underlying differences between men and women.

Our study has several limitations. The limited sample size prevented us from exploring the independent association of gender and HIV risk behaviors in a robust multivariate model. Also, our convenience sampling strategy reduces the generalizability of our study to all recently-released individuals. Our survey did not ask about other factors that impact HIV risk taking behavior, such as physical and sexual abuse as well as mental health history. Finally, our study used a well-established measure of AIDS knowledge that was designed prior to the advent of HAART, perhaps limiting its accuracy.

## Conclusion

Our findings may suggest the need for interventions targeting women focused on reducing drug addiction and providing employment opportunities that counter the financial incentives for engaging in transactional sex. Larger, longitudinal studies are needed to better understand the gender differences in HIV risk behavior engagement in this population.
